# Single nucleotide polymorphisms at the *TRAF1/C5 *locus are associated with rheumatoid arthritis in a Han Chinese population

**DOI:** 10.1186/1471-2350-12-53

**Published:** 2011-04-14

**Authors:** Jing Zhu, Dinging Zhang, Fengxia Wu, Fei He, Xiaoqi Liu, Lijun Wu, Bin Zhou, Jianping Liu, Fang Lu, Jian Liu, Ruijun Luo, Wubin Long, Minghui Yang, Shi Ma, Xiaodan Wu, Yi Shi, Tong Wu, Ying Lin, Jiyun Yang, Guohua Yuan, Zhenglin Yang

**Affiliations:** 1 Department of Rheumatology and Immunology, Sichuan Academy of Medical Sciences & Sichuan Provincial People's Hospital, Chengdu, Sichuan, China; 2 Center for Human Molecular Biology & Genetics, Sichuan Academy of Medical Sciences & Sichuan Provincial People's Hospital, Chengdu, Sichuan, China; 3 Institute of Laboratory Medicine, Sichuan Academy of Medical Sciences & Sichuan Provincial People's Hospital, Chengdu, Sichuan, China; 4 Institute of Rheumatology and Immunology, Affiliated Hospital, North Sichuan Medical College, Nanchong, Sichuan, China; 5 Department of Rheumatology, People's Hospital of Xin Jiang Uygur Autonomous Region, Urumchi, Xin Jiang, China

**Keywords:** rheumatoid arthritis, genetics, *TRAF1/C5*, association study, Chinese

## Abstract

**Background:**

Genetic variants in *TRAF1C5 *and *PTPN22 *genes have been shown to be significantly associated with arthritis rheumatoid in Caucasian populations. This study investigated the association between single nucleotide polymorphisms (SNPs) in *TRAF1/C5 *and *PTPN22 *genes and rheumatoid arthritis (RA) in a Han Chinese population. We genotyped SNPs rs3761847 and rs7021206 at the *TRAF1/C5 *locus and rs2476601 SNP in the *PTPN22 *gene in a Han Chinese cohort composed of 576 patients with RA and 689 controls. The concentrations of anti-cyclic citrullinated peptide antibodies (CCP) and rheumatoid factor (RF) were determined for all affected patients. The difference between the cases and the controls was compared using *χ*^2 ^analysis.

**Results:**

Significant differences in SNPs rs3761847 and rs7021206 at *TRAF1/C5 *were observed between the case and control groups in this cohort; the allelic p-value was 0.0018 with an odds ratio of 1.28 for rs3761847 and 0.005 with an odds ratio of 1.27 for rs7021206. This significant association between rs3761847 and RA was independent of the concentrations of anti-CCP and RF. No polymorphism of rs2476601 was observed in this cohort.

**Conclusions:**

We first demonstrated that genetic variants at the *TRAF1/C5 *locus are significantly associated with RA in Han Chinese, suggesting that *TRAF1/C5 *may play a role in the development of RA in this population, which expands the pathogenesis role of *TRAF1/C5 *in a different ethnicity.

## Background

Rheumatoid arthritis (RA) is a chronic inflammatory disease that affects about 1% of the adult Caucasian population and 0.37% of the Chinese population. Women are affected three times more often than men [1-3]. This disease may affect many tissues and organs; it particularly destroys synovial joints involving autoimmune features [2]. The disease can cause severe disability and even early mortality.

Although the full etiology of RA remains unclear, it is considered a complex disease caused by the interaction of genetic variants, the environment, infectious and hormonal factors [4]. The genetic variants may contribute 50-60% of the etiology of RA [5]. At least 31 RA risk loci have been confirmed associated with RA in different populations [6-14]. At *TRAF1/C5 *locus, both TRAF1 and C5 are possible RA-causing genes due to their biological functions. TNF is a critical cytokine in the pathogenesis of RA [15]. TRAF1 and TRAF2 form a heterodimeric complex, which is required for TNF-alpha-mediated activation of MAPK8/JNK and NF-kappaB. The protein complex formed by this protein and TRAF2 also interacts with inhibitor-of-apoptosis proteins (IAPs), and thus mediates the anti-apoptotic signals from TNF receptors. TNF antagonists are an effective treatment for rheumatoid arthritis [15-17]. On the other hand, the clinical and biological data for C5 are equally compelling [15]. The complement pathway has been implicated in the pathogenesis of RA for a long time [18,19]. Complement activation leading to significant depletion of complement components has been shown in synovial fluid of patients with RA [15]. rs7021206 is located at TRAF1 intron 3 and rs3761847 is located at the upstream of TRAF1 and the down stream of C5. These variants or other causative variants at this locus may affect the function or expression levels of TRAF1 and/or C5 to lead to RA.

In this study, we investigated the association between rs7021206 and rs3761847 at the *TRAF1/C5 *locus and rs2476601 in the *PTPN22 *gene and RA in a Han Chinese population.

## Methods

### Patients

The Institutional Review Boards of the Sichuan Academy of Medical Sciences & Provincial People's Hospital, North Sichuan Medical College, China, approved this study. All subjects provided informed consent before participating in the study. RA patients and normal matched controls, including individuals with a normal joint examination, were recruited at the rheumatology and Immunology clinics at Sichuan Provincial People's Hospital and North Sichuan Medical College, China. All participants went through a standard examination protocol as noted in the previous description: (http://medcalc3000.com/RheumatoidArthritis.htm, Rheumatoid Arthritis Criteria 1987 revision, American Rheumatism Association), including morning stiffness, arthritis of three or more joints, including hand joints, symmetric arthritis, rheumatoid nodules, serum rheumatoid factor, and radiographic changes. In total, 576 patients with RA and 689 normal age-matched controls were recruited. All patients enrolled in this study at least two years after clinical diagnosis of RA and the patients had five years of mean disease duration. In the normal controls, no sign of joint or other autoimmune disease was detected. Clinical information about the cases and controls is listed in Table 1.

**Table 1:** Characteristics of RA cases and controls matched for ages and ethnicity

Subject	Total number	anti-CCP positive	RF positive	Male	Female	Average age
Cases	576	363	496	266	310	64 ± 6.6
Controls	689	0	0	307	382	65 ± 7.8

± represents standard deviation

### CCP and RF measurement

Sera were obtained and tested for concentrations of RF and anti-CCP in all patients. The concentrations of anti-CCP antibodies were detected with an Anti-CCP-ELISA kit (EUROIMMUN Medizinische Labordiagnostika AG, Lübeck, Germany) on a Bio Rad Bench Mark machine (Rio Rad, Hercules, CA, USA). RF was determined with an endpoint nephelometry kit (Siemens Healthcare Diagnostics Products GmbH, Marburg, Germany) on a DADE BEHRING BNII machine (GMI, Minnesota, USA) according to the manufacturer's manual instructions. Results were corroborated by validation at the clinical laboratory of Sichuan Provincial People's Hospital, Sichuan, China. Cases with anti-CCP levels higher than 20units/ml were considered positive for anti-CCP antibodies; cases with RF levels higher than 5 units/ml were considered positive for RF.

### Genotyping

Based on previous genome-wide association studies (GWAS) and replication studies in Asians [6-13,20], we selected rs7021206 and rs3761847 at the *TRAF1/C5 *locus and rs2476601 in the *PTPN22 *gene to genotype in the Han Chinese population. Venous blood from each subject was withdrawn and collected in an EDTA tube. Genomic DNA was extracted from the blood by serial phenol/chloroform extraction and ethanol precipitation. SNP genotyping was performed with the dye terminator-based SNaPshot method (Applied Biosystems, CA, USA). The SNP reported in this manuscript has a genotyping success rate of 97% and accuracy as judged by random re-genotyping of 10% of the samples in the cohort. For rs3761847, the PCR forward primer 5'-CCTACCTGTTCCCTCCTTCC-3', PCR reverse primer 5'-GGGATGATGATGGCAATACC-3', and SNaPshot primer 5'- AGGTAGAGAGGGTGGTATTGAGGC-3' were used in genotyping. For rs7021206, the PCR forward primer 5'-GAGAAGCAGATGGGAGAGTG-3', PCR reverse primer 5'-TGCTTGGCTAGCAGCAATTC-3', and SNaPshot primer 5'-AAGGAGAAGGAGGTGTGGACACTCGCCTGCCTCTGGTCCA-3' were used in genotyping. For rs2476601, the PCR forward primer 5'- GGCCTCAATGAACTCCTCAA-3', PCR reverse primer 5'- GGATAGCAACTGCTCCAAGG-3'and SNaPshot primer 5'-CCTCAACCACAATAAATGATTCAGGTGTCC-3' were used in genotyping.

### Statistical analysis

The Hardy-Weinberg equilibrium (HWE) for the SNP was tested with the χ^2 ^test. All analyses were adjusted for matching factors of age and sex factors. Allele and genotype frequencies between cases and controls were compared with the χ^2 ^analysis. Statistical significance was defined as p < 0.05. All statistical analyses were performed using the software SPSS version 10.0.

## Results

Among the 576 patients with RA, 363 of them were anti-CCP positive and 496 of them were RF positive (Table 1). Linkage disequlibrium between rs3761847 and rs7021206 was 0.23 (r^2^) in the Chinese cohort studied. Allele distributions both of rs3761847 and rs7021206 were within the Hardy-Weinberg equilibrium in both the case and control groups (p > 0.05, Table 2). We found that both rs3761847 and rs7021206 were significantly associated with RA in the cohort studied (Allelic p = 0.0018, adjusted p = 0.054 for rs3761847; allelic p = 0.005, adjusted p = 0.015 for rs7021206, Table 2). For rs3761847, the risk allele frequency was 0.58 in the patients and 0.52 in the controls. The risk allele of this SNP conferred a 1.28-fold (95% CI: 1.10~1.50) increased likelihood of RA (Table 2). For rs7021206, the risk allele frequency was 0.34 in the patients and 0.29 in the controls. The risk allele of this SNP conferred a 1.27-fold (95% CI: 1.07~1.50) increased likelihood of RA (Table 2). rs3761847 showed significant association with RA in both the anti-CCP positive and anti-CCP negative groups. The p value is 0.0015 in the anti-CCP positive group with an odds ratio (OR) of 1.33 (95% CI: 1.11-1.60; Table 3); the p value is 0.043 in the anti-CCP negative group with an OR of 1.25 (95% CI: 1.0-1.56; Table 3). rs3761847 also showed a significant association with RA in both the RF positive and negative groups. The p value was 0.0015 in the RF positive group with an OR of 1.30 (95% CI: 1.10-1.53; Table 3); the p value was 0.03 in the RF negative group with an OR of 1.44 (95% CI: 1.03-2.01; Table 3). rs7021206 also showed significant association with RA in both the anti-CCP positive and RF positive groups, (p = 2.5 × 10^-4 ^and 1.4 × 10^-4^, respectively, table 3), but not in anti-CCP or RF negative groups (p = 0.065, 0.29, respectively, table 3).

**Table 2:** Association between rs3761847 and rs7021206 in TRAF1/C5 and RA in a Han Chinese cohort

SNP	Phenotype	HWE	Genotype count (frequency)	Allele frequency	Allelic *p*	Adjusted p	OR (95%CI)
	Case (576)	0.93	AA: 192 (0.33)	A:0.58	0.0018	0.0054	1.28 (1.10-1.50)
			AG: 282 (0.49)	G:0.42			
rs3761847			GG: 102 (0.18)				
	Control (689)	0.06	AA: 171 ( 0.25)	A:0.52			
			AG: 369 ( 0.54)	G:0.48			
			GG:149 ( 0.21)				

	Case (576)	0.07	AA: 239 (0.41)	A:0.66	0.005	0.015	1.27 (1.07-1.50)
			AG: 279 (0.48)	G:0.34			
rs7021206			GG: 58 (0.11)				
	Control (689)	0.54	AA: 343 (0.50 )	A:0.71			
			AG: 291 (0.42 )	G:0.29			
			GG: 55 ( 0.08 )				

SNP: single nucleotide polymorphism, HWE: Hardy-Weinberg equilibrium, OR: odds ratio, CI: confidence interval.

**Table 3:** Association of rs3761847 and rs7021206 in TRAF1/C5 and subgroup RA in a Han Chinese cohort

SNP	*Group*	*HWE*	*Genotype count and frequency*		*Allele frequency*		*Allelic p*	*OR (95%CI)*
					
			*Case (frequency)*	*Control (frequency)*	*Case*	*Control*		
	*anti-CCP positive*	*Case: 0.76*	*AA:127 (0.35)*	*AA:171 ( 0.25)*	*A: 0.59*	*A: 0.52*	*0.0015*	*1.33 (1.11-1.60)*
		*Control: 0.058*	*AG:173 (0.48)*	*AG:369 ( 0.54)*	*G: 0.41*	*G: 0.48*		
			*GG:63 (0.17)*	*GG:149 ( 0.21)*				
	*anti-CCP negative*	*Case: 0.81*	*AA:69 (0.32)*	*AA:171 ( 0.25)*	*A: 0.57*	*A: 0.52*	*0.040*	*1.25 (1.01-1.56)*
		*Control: 0.058*	*AG:106 (0.50)*	*AG:369 ( 0.54)*	*G: 0.43*	*G: 0.48*		
rs3761847			*GG:38 (0.18)*	*GG:149 ( 0.21)*				
	*RF positive*	*Case: 0.81*	*AA:170 (0.34)*	*AA:171 ( 0.25)*	*A: 0.58*	*A: 0.52*	*0.0015*	*1.30 (1.10-1.53)*
		*Control: 0.058*	*AG:237 (0.48)*	*AG:369 ( 0.54)*	*G: 0.42*	*G: 0.48*		
			*GG:89 (0.18)*	*GG:149 ( 0.21)*				
	*RF negative*	*Case: 0.26*	*AA:27 (0.34)*	*AA:171 ( 0.25)*	*A: 0.61*	*A: 0.52*	*0.030*	*1.44 (1.03-2.01)*
		*Control: 0.058*	*AG:43 (0.54)*	*AG:369 ( 0.54)*	*G: 0.39*	*G: 0.48*		
			*GG:10 (0.16)*	*GG:149 ( 0.21)*				

	*anti-CCP positive*	*Case: 0.22*	*AA:139 (0.38 )*	*AA:343 (0.50 )*	*G: 0.37*	*G: 0.29*	*0.00025*	*1.42 (1.18-1.72)*
		*Control: 0.54*	*AG:180 (0.50 )*	*AG:291 (0.42 )*	*A: 0.63*	*A: 0.71*		
			*GG:44 (0.12 )*	*GG:55 ( 0.08 )*				
	*anti-CCP negative*	*Case: 0.18*	*AA:89 (0.42 )*	*AA:343 (0.50 )*	*G: 34*	*G: 0.29*	*0.065*	
		*Control: 0.54*	*AG:104 (0.49 )*	*AG:291 (0.42 )*	*A: 0.66*	*A: 0.71*		
rs7021206			*GG:20 (0.09 )*	*GG:55 ( 0.08 )*				
	*RF positive*	*Case: 0.17*	*AA:193 (0.39 )*	*AA:343 (0.50 )*	*G: 0.36*	*G: 0.29*	*0.00014*	*1.40 (1.18-1.67)*
		*Control: 0.54*	*AG:244 (0.49 )*	*AG:291 (0.42 )*	*A: 0.64*	*A: 0.71*		
			*GG:59 (0.12 )*	*GG:55 ( 0.08 )*				
	*RF negative*	*Case: 0.26*	*AA:38 (0.48)*	*AA:343 (0.50 )*	*G: 0.33*	*G: 0.29*	*0.29*	
		*Control: 0.54*	*AG:31 (0.39)*	*AG:291 (0.42 )*	*A: 0.67*	*A: 0.71*		
			*GG:11 ( 0.13 )*	*GG:55 ( 0.08 )*				

SNP: single nucleotide polymorphism, HWE: Hardy-Weinberg equilibrium, OR: odds ratio, CI: confidence interval.

No polymorphism was observed for rs2476601 in the cohort.

## Discussion

Although at least 31 susceptibility loci/genes responsible for RA have been identified [14]; including *HLA-DRB1 *([7,13], *protein tyrosine phosphatase 22 *(*PTPN22*) gene [13,21], *tumor necrosis factor, alpha-induced protein 3 *(*TNFAIP3*) locus on chromosome 6q [8,9,13], *TRAF1/C5 *[10,11], *STAT4 *[12], *interferon regulatory factor 5 isoform a *(*IRF5*) [22], peptidylarginine deiminase (*PADI4*) [23], *CD244 natural killer cell receptor 2B4 *(*CD244*) [24], *CD40 antigen isoform 2 precursor *(*CD40*) [25], *small inducible cytokine A21 precursor *(CCL21) [25], and *solute carrier family 22 member 4 *(*SLC22A4*) [26] and others [14], only 5 loci/genes (*HLA, PTPN22, TRAF1/C5, TNFAIP3*, and *STAT4*) have been successfully replicated in different ethnicities, whereas others have been observed less systematically or further replication studies in different ethnicities are needed. Among the five successfully replicated loci, *HLA *showed a significant association with RA in all different ethnicities. The association of *STAT4 *with RA has also been successfully replicated in Asians, including Japanese, Koreans and Chinese [27-29]; and the association of *TRAF1/C5 *with RA has also been replicated in Japanese and Korean populations [20,30].

Although rs2476601 (R620W) in *PTPN22 *has been constantly associated with RA in populations of European descent, including US [31], UK [32], Finnish [33], Swedish [34], German [35], Dutch [36], Spanish [37], and Canadian [38] populations, this SNP did not show a significant association with RA in the Japanese population [39] studied because there is a very low minor allele frequency in this population. In addition, although tag SNPs covering the *PTPN22 *linkage disequilibrium block were polymorphic in the Korean population, Lee et al did not reveal any disease association, and the re-sequencing did not identify any new common coding region variants in this population [40]. Consistent with results in Japanese and Korean populations, we did not find even a single minor allele of rs2476601 in the studied Chinese cohort, suggesting that rs2476601 in *PTPN22 *is not associated with RA in Asians.

On the other hand, genetic variants in *PADI4 *have shown significant association with RA in Asian populations [24,41,42] but not in Caucasian populations [34,43]. Therefore, we believe the genetics of RA shows obvious ethnic differences.

rs3761847 at the *TRAF1/C5 *locus has been significantly associated with RA in an initial genome-wide association study (GWAS) in a North American and Swedish cohort, composed of 1493 cases and 1831 controls [11]. This finding was replicated in the second cohort composed of 485 cases and 1282 controls in a North American population [11]. In addition, using a candidate gene approach, Kurreeman et al identified that rs10818488 in *TRAF1/C5 *is significantly associated with RA in patients of Dutch origin [10]. This finding was confirmed in a case-control study in the population of the island of Crete, Greece [44] and a meta-analysis [45]. Kurreeman et al also confirmed the *TRAF1/C5 *locus as a susceptibility locus for rheumatoid arthritis in a European family-based replication study using rs10818488 at this region [46]. However, this association could not be confirmed in a Swedish cohort composed of 568 cases and 516 controls. Chang et al [15], carried out a multi-tiered, case-controlled association study, genotyping 25,966 putative functional SNPs in an initial North America cohort, composed of 475 cases and 475 controls and two replication cohorts (661 cases/1322 controls from North America and 596 cases/705 controls from the Netherlands). The authors identified an SNP, rs1953126 at the *TRAF1/C5 *locus that was significantly associated with RA (OR = 1.28, trend p = 1.45 × 10^-6^). Through a comprehensive fine-scale-mapping study, they found a variety of analyses identified SNPs in a 70 kb region extending from the third intron of *PHF19 *across *TRAF1 *into the *TRAF1-C5 *intergenic region, excluding the *C5 *coding region. This study suggested that *TRAF1 *is the RA gene at the *TRAF1-C5 *locus, but further studies are needed to confirm this conclusion.

In recent studies, rs3761847 has been shown to be significantly associated with RA in Japanese [30]. Our results further confirmed that rs3761847 at the *TRAF1/C5 *was associated with RA in Han Chinese Asians, and this association might not depend on the concentrations of anti-CCP antibodies or RF. Thus, TRAF1/C5 is another locus playing a role in RA development in different ethnicities. We observed that rs3761847 A increased the risk for RA in the present study, which is consistent with the results observed in a Japanese population [30], however, this is the opposite of the original studies in Caucasian populations, in which rs3761847 A decreased the risk of RA. In addition, rs3761847 did not show a significant association with RA in the Korean population [20,30]. On the other hand, we found that rs7021206 at intron 3 of TRAF1 was showed significant association with RA in the Han Chinese in this study, which is consistent with previous studies in Korean and Caucasian populations [20]. This indicated that rs7021206, but not rs376184, may be the true causative allele at the TRAF1-C5 locus [20,47]; However, the true causative allele in this region needs to be further investigated.

The significant association between rs3761847 and RA was observed in both anti-CCP and RF positive groups and negative groups, suggesting that this significant association between TRAF1-C1 and RA was independent of the concentrations of anti-CCP and RF [20]. No significant association was observed between rs7021206 and RA in anti-CCP and RF negative groups; this may be because of small sample size in these groups in the current studies.

Other studies indicate genetic variants at the *TRAF1/C5 *locus are associated with autoimmune diseases affecting multiple organs, such as systemic lupus erythematous (SLE) [48,49], and juvenile idiopathic arthritis [50-52]. Thus, the functional study of TRAF1/C5 with autoimmune disease may provide an insight into the pathogenesis and treatment of these diseases as well as RA.

## Conclusions

We first demonstrated that genetic variants rs3761847 and rs7021206 in *TRAF1/C5 *locus are significantly associated with RA in the Han Chinese, suggesting that *TRAF1/C5 *may play a role in the development RA in this population, which expands the pathogenesis role of *TRAF1/C5 *in a different ethnicity. Unlike in Caucasian populations, rs2476601 in the *PTPN22 *gene did not show polymorphism in the Han Chinese population being studied, suggesting that either other genetic variants in this gene are associated with RA in the Han Chinese population or *PTPN22 *has nothing to do with RA the population.

## Competing interests

The authors declare that they have no competing interests.

## Authors' contributions

Designed the study: ZY and JZ; collected samples and experiments: JZ, DZ, FW, LW, BZ, JL, FL, JL, RL, WL, MY, SM, XW, YS, TW, YL, JY, GY and ZY; performed the data analysis: XL and ZY; writing the manuscript: ZY. All authors read and approved the final manuscript.

## Pre-publication history

The pre-publication history for this paper can be accessed here:

http://www.biomedcentral.com/1471-2350/12/53/prepub
